# Sex and rhythms in sandflies and mosquitoes: An appreciation of the work of Alexandre Afranio Peixoto (1963–2013)

**DOI:** 10.1016/j.meegid.2014.06.016

**Published:** 2014-12

**Authors:** Charalambos P. Kyriacou

**Affiliations:** Department of Genetics, University of Leicester, Leicester Le1 7RH, UK

**Keywords:** Sandflies, Mosquitoes, Behaviour, Copulation songs, Population genetics, Phylogeny

## Abstract

I will briefly discuss the work of Alexandre A. Peixoto on sandflies and mosquitoes, focusing initially on his contributions to the population biology and phylogenetics of Brazilian populations of these important hematophagous insects. I shall also review some of his work on the underlying molecular clocks that mediate rhythmic behaviour and physiology in these species.

## Highlights

• *Lutzomyia longipalpis* in Brazil has recently undergone complex speciation events.
• *Anopheles cruzii* in southern Brazil has also undergone recent speciation.
• The circadian clock mechanisms of both sandflies and mosquitoes have been described.

## 1 Introduction

Alexandre Peixoto began his research career in Rio de Janeiro as a Masters student working on the population genetics and molecular evolution of chromosomal inversion polymorphisms in *Drosophila melanogaster*. He subsequently won a CNPq scholarship to work at the University of Leicester’s Genetics department where he landed in the late 1980’s to work in my laboratory. Here he learned molecular biology and extended his evolutionary skills to the field of *Drosophila* circadian rhythms. He was to take the circadian rhythms project into insect vectors when he returned to Brazil some years later, but after Leicester, he secured a postdoctoral period in Jeff Hall’s laboratory at Brandeis University in the USA, where he worked on Drosophila courtship songs. Again, this theme was to resurface in the context of insect vectors once he took up his position at the Oswaldo Cruz Institute in Rio. His work on Drosophila circadian rhythms has been reviewed many times so I will refrain from going over the old literature (Kyriacou et al., 2008). Rather I would like to review his behavioural genetic and evolutionary work on sandflies and mosquitoes, which from 2001 onwards, generated more than 60 publications. One can divide Alex’s work as a principal investigator into three overlapping categories which reflected his phylogenetic and behavioural interests in sandflies, then in mosquitoes, then finally in his gene expression work on circadian clocks in the two hematophagous insects. I shall take each of these in order and highlight his major contributions.

## 2 Cryptic speciation in sandflies of the Americas

In the first approach, he took the view that in the sandfly, *Lutzomyia longipalpis*, which carries leishmaniasis in the Americas, the possibility of recent speciation in central and South American population could have important implications for vectorial capacity. To best study such populations, he combined behavioural and phylogenetic methods. Alex had previously worked on clock and song genes in Drosophila, and it was known that such behavioural genes evolved rapidly (Colot et al., 1988). This was because genes like *period* which was originally identified by mutagenesis for circadian clock phenotypes (Konopka and Benzer, 1971), had been shown to act as a reservoir for species-specific timing information that was important for mate recognition, temporal isolation and assortative mating (Petersen et al., 1988, Wheeler et al., 1991, Tauber et al., 2003). Behaviour is a phenotype that can reliably distinguish species when anatomical characteristics cannot, a feature well known to the early ethologists. Consequently a gene like *period*, that determines biological timing over a wide range of time domains in Drosophila, and which could conceivably contribute to speciation, might, like rapidly evolving mitochondrial DNA, be just the type of gene sequence that could be used to distinguish incipient species.

**Fig. 1:**
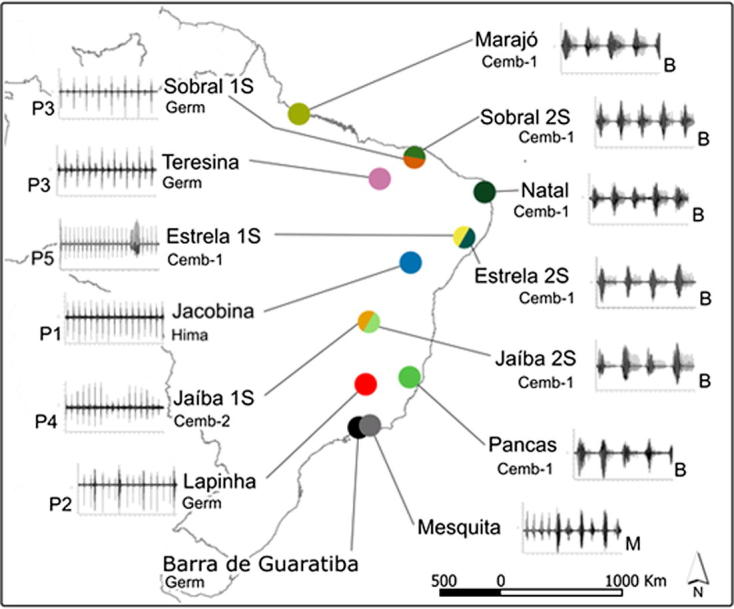
Courtship songs of *Lutzomyia longipalpis* populations from Brazil (from Araki et al., 2009).

With this in mind, Alex began to study the courtship songs of the male sandfly in a number of Brazilian populations (Souza et al., 2004, Araki et al., 2009)). The male’s song was produced by wing vibration, but unlike *D. melanogaster*, it is generated during copulation and not during courtship. Initially two different types of songs were observed in a number of populations pulse (P) and burst (B) song (Souza et al., 2004). This was later expanded to three song types when a population from Mesquita was studied which had a mixed (M) pulse-burst type of song (Fig. 1, Araki et al., 2009). It was the pulse-type song that seemed to show the most variation among populations with five different patterns distinguished (P1–P5, Fig. 1), each potentially identifying a separate incipient species. Indeed, when males and females from different populations were mixed together, copulation frequency was reduced and none of the females produced larvae (Souza et al., 2008). This was observed among three allopatric populations (Jacobina, Lapinha and Natal, Fig. 1) and was even more pronounced between two sympatric populations from Sobral suggesting reinforcement of pre-mating isolation (Sobral 1S and 2S, Fig. 1). Thus differences among the song types were associated with reproductive isolation, suggesting the existence of a species complex.

Other populations, Teresina, Barra de Guaratiba, and Pancas that showed different song patterns also had different pheromonal profiles (Fig. 1, Araki et al., 2009). In addition, sequence analysis of a fragment of the *period (per)* circadian clock gene revealed that in pairwise F_ST_ tests, significant subdivision was observed in 42/51 comparisons that were spread equally between pairs of pulse song and pulse-burst song populations. In contrast, the same analyses between pairs of burst type populations revealed only one out of 15 significant F_ST_ values. Consequently populations that produce bursts are more similar genetically to each other and express a similar pheromone, cembrene-1, whereas populations that produce the different subtypes of pulses are less related to each other and to burst song populations and carry a more heterogeneous blend of pheromones (Araki et al., 2009). In the three sets of sympatric populations Sobral, Estrela and Jaiba (Fig. 1), there was always a song difference between each pair or populations, in some there was also a pheromone difference, and always a significant F_ST_ so it may well be that character differences are amplified in sympatry (see below). What is clear is that *L. longipalpis* represents a species complex with possibly 5 incipient pulse-type species as well as a more homogeneous burst-type song species.

A subsequent multilocus analysis of 21 different genes from the sympatric species from Sobral, and the allopatric ones from Lapinha and Pancas revealed greater differentiation using F_ST_ tests between the allopatric than the sympatric pair of species in 19/21 loci (Araki et al., 2013). This result suggested that a higher level of introgression was occurring between the sympatric pair in spite of the fact that Pancas and Lapinha, in terms of song and pheromone, were more similar to Sobral 2S and Sobral 1S respectively (IS and 2S refers to the number of pale paired spots on the abdomen, Fig. 1). An overall picture emerges from this comprehensive analysis of a number of population genetics parameters, that about 0.5 Mya, the two pulse/burst pheromone lineages separated, then came into secondary contact in Sobral leading to introgression because reproductive isolation was not complete. However, as other characters among the sympatric species such as locomotor rhythms (Rivas et al., 2008)and developmental characteristics also differ(Souza et al., 2009), there is a suggestion that character displacement might be occurring, which along with song differences, reinforces the mating barriers and separation of the species (Araki et al., 2013). Introgression between other *Lutzomyia* species, *Lutzomyia whitmani* and *Lutzomyia intermedia* has also been detected using a similar multilocus approach (Mazzoni et al., 2006, Mazzoni et al., 2008). Gene flow between incipient species could represent one way that sequences that might have relevance for transmission of the *Leishmania* parasite can be spread. However, it still remains to be established whether these sibling species differ in their vectorial capacity.

## 3 Speciation in *Anopheles cruzii*

Alex began his mosquito work examining reproductive isolation and the role of the X chromosome in hybrids of the two sibling species of Anopheles, *Anopheles albitarsis* and *Anopheles deneorum*, both malarial vectors (Lima et al., 2004). However he graduated to *An. cruzii*, an important carrier of Plasmodium in southern Brazil, and, as he did with sandflies, he used population genetics statistics to investigate cryptic speciation. In his first study he used a fragment of the *timeless* clock gene and identified at least two species within the complex, one found in Bahia, a little further north than the other. He roughly estimated that the population in Bahia had diverged about 1.5 Mya from five, more southern populations (Rona et al., 2009). He then used a multilocus approach with sequences from three clock and three ribosomal protein genes and arrived at a variety of divergence times ranging from 1.1 to 3.6 My, whilst observing very high F_ST_ values, suggesting that there had been very little introgression since the two species had separated (Rona et al., 2010, Rona et al., 2010).

He also reinvestigated the five more southern populations using sequences from the NAPDH-cytochrome P450 reductase gene and found evidence for further cryptic speciation within one of these localities (Rona et al., 2010, Rona et al., 2010). Using the same six genes and a multilocus approach he estimated that these sympatric populations, while showing some asymmetric introgression, had nevertheless diverged approximately 0.19 Mya, representing a case of incipient speciation. In turn, these sympatric populations had diverged about 0.75 My from the other allopatric populations (Rona et al., 2013). Thus we can see, there have been three main speciation events for *An. cruzii* in this part of Brazil, the first dividing the more northern from the southern species more than 1 Mya, followed by a latter speciation in the south about 0.75 Mya, and then an incipient speciation in a smaller southern locality region 0.2 Mya. Like sandflies, *An. cruzii* show a complex pattern of recent speciation events.

## 4 Clock gene expression in sandflies and mosquitoes

The third main thrust of Alex’s work was in the comparative analysis of clock gene expression in hematophagous insects. Almost all insect behaviour is modulated by the circadian clock, and activity and biting of hematophagous insects are no exception. Consequently, Alex was keen to investigate circadian clock gene expression in both sandflies and mosquitoes. He examined the locomotor patterns of *L. longipalpis* females and observed that unlike *D. melanogaster*, they did not show a very prominent morning (M) peak of activity but did show the evening peak (E) that anticipates the lights off signal (Meireles-Filho et al., 2006b). He also revealed that the sympatric species of *L. longipalpis* from Sobral showed slightly different phases in locomotor activity rhythms (Rivas et al., 2008). A slight reduction of locomotor activity of the E component in blood-fed females was also observed (Meireles-Filho et al., 2006b).

In Drosophila, the intracellular mechanism that we call the circadian clock is represented by a set of interconnected molecular feedback loops (reviewed in (Ozkaya and Rosato, 2012)). The most studied loop has two negative regulators, PERIOD (PER) and TIMELESS (TIM), two positive regulators, CLOCK (CLK) and CYCLE (CYC), that act as a dimer to transcribe *per* and *tim* genes, a series of modulators that include kinases and phosphatases that regulate the stability of negative (and also positive) regulators, and a photoreceptor, CRYPTOCHROME (CRY). Early at night CLK/CYC transcribe *per* and *tim* mRNAs are but as PER is translated, it is phosphorylated and degraded. Later at night, when TIM levels build up, it dimerises with PER and protects it from phosphorylation. TIM and PER then enter the nucleus late at night and sequester the CLK-CYC dimer, thereby repressing their own transcription. Early the next day, PER and TIM degrade so *per/tim* transcription is de-repressed and another cycle begins. PER-TIM degradation around dawn-time will occur in darkness, but in a light –dark cycle, CRY is activated by light and that also leads to TIM and then PER degradation followed by *per/tim* derepression. Two further feedback loops involving CLK and an exotically named CLOCXWORK ORANGE (CWO) also intersect the PER/TIM loop and stabilise the transcriptional/translational oscillation. The net result of all this activity is that several of the main components cycle at both mRNA and protein levels (Ozkaya and Rosato, 2012).

Fragments of *per, tim and Clk* were isolated from *L. longipalpis*, and mRNA rhythms were observed in all three transcripts from the head with a peak just after lights-off. In the female body however, *per* did not cycle (as is also observe in *D. melanogaster* females) but *tim* and *Clk* cycled in antiphase. After a blood meal, there was a reduction of *per* and *tim* mRNA levels in both heads and bodies which correlated with reduced E activity (Meireles-Filho et al., 2006b). Unlike *D. melanogaster* however, the *cycle (cyc)* transcript was also expressed rhythmically in male and female heads (Meireles-Filho et al., 2006a) and found to be at higher levels early in the day in several brain areas that correspond to clock gene expression in Drosophilids (Chahad-Ehlers et al., 2013). The CYC protein contained an activation sequence that is also observed in the mammalian orthologue BMAL1 as well as in some other insect CYCs, but not in the *Drosophila* sequence, in which CLK carries the activation domain (Allada et al., 1998). Blood-feeding had no effect on *cyc* expression, so the effect of blood feeding appears limited to the negative regulators *per* and *tim* in the sandfly. In contrast however, in *Aedes aegypti*, the vector of dengue and yellow fever, blood feeding led to a dramatic downregulation of both negative (*per/tim*) and positive (*Clk/cyc*) factors in the body, with a gentler reduction in the head (Gentile et al., 2013).

*A. aegypti* was also compared to *Culex quinquefasciatus* both in terms of circadian behavioural and molecular rhythms. Behaviourally, *Aedes* is diurnal whereas *Culex* is nocturnal (Gentile et al., 2009). Molecular analysis of the two negative and two positive regulators, as well as two regulators of the CLK loop *Pdp1ε* and *vrille*, revealed that all of these mRNAs cycled in both species, but the profiles were similar. Both species of mosquito encode two *cry* genes, the photoreceptor-like Drosophila *cry* type 1 but also the mammalian *cry* type 2. Mammals have two copies of type 2 CRYs, which act as the main negative regulators of the clock, replacing the role of the negative factor TIM in the fly (Clayton et al., 2001). Several insects also encode CRY type 2 molecules which act as negative regulators (Yuan et al., 2007). Very intriguingly, the rhythmic *cry2* mRNA profiles were dramatically different between the two species, both in light-dark cycles and in constant darkness. In *Culex*, the normal single peak of cycling mRNA was observed, as one would expect of negative regulators in insects, with a peak early at night and in the same phase as *per* and *tim* in both mosquitoes. However at the beginning of the day, there was an additional striking mRNA peak in *Aedes* (Gentile et al., 2009).

Of course the correlation between being diurnal or nocturnal may have nothing to do with this difference in clock gene regulation between the two species. One would need interspecific molecular replacements of the genes and their promoters allied to behavioural analysis to investigate whether these two phenotypes are causally related. This is technically impossible to do with current transformation technology. Yet there is some further data on an agricultural pest species, the melon fly, *Bactrocera cucurbitae*, that cyclical *cry* expression is also different between two strains that differ in circadian period and daily mating times (Fuchikawa et al., 2010). In two other *Bactrocera* species, *Bactrocera neohumeralis* and *Bactrocera tryoni*, *cry* type 1 levels cycle in the brain and antennae, but there are significantly higher levels in the former than the latter which correlate with the enhanced mating propensity of *B. tryoni* at dusk (An et al., 2004). In hybrids selected for early or late mating, similarly altered levels of *cry* were observed in the dusk-mating line, so it seems reasonable to propose that there is more than just a spurious correlation between *cry* levels and mating times (An et al., 2004).

## 5 Conclusions

I have attempted to cover some of the more prominent aspects of Alex’s work and I hope that the reader can see the rationale that drove his studies. Understanding the speciation of these important insect vectors could underlie any differential ability that may exist among the sibling species in disease transmission. It would thus be a fitting conclusion to Alex’s work if the vectorial capacity of these cryptic species of sandflies and mosquitoes was studied further. Secondly, circadian rhythms are so fundamentally important for all aspects of insect physiology and behaviour that their study may reveal chinks in the armour of these important hematophagous insects that might be exploited in future. It is my hope that somebody will pick up the baton from Alex and consolidate and extend his pioneering work in this area of medically-related entomology.
